# Evaluation of chromID strepto B as a screening media for *Streptococcus agalactiae*

**DOI:** 10.1186/1471-2334-14-46

**Published:** 2014-01-29

**Authors:** Toyohisa Morita, Dongyun Feng, Yoko Kamio, Isao Kanno, Teruo Somaya, Kazuhito Imai, Misaki Inoue, Mutsunori Fujiwara, Akihito Miyauchi

**Affiliations:** 1 Department of Laboratory Medicine, Division of Clinical Microbiology, Japanese Red Cross Medical Center, 4-1-22 Hiroo, Shibuya-ku, Tokyo 150-8935, Japan; 2 Department of Scientific Research, Division of Scientific Affairs, Sysmex Corporation, 1-3-2 Murotani, Nishi-ku, Kobe 651-2241, Japan; 3 Department of Obstetrics and Gynecology, Japanese Red Cross Medical Center, Shibuya-ku, Tokyo, Japan

**Keywords:** Group B Streptococci, Perinatal screening, *Streptococcus agalactiae*

## Abstract

**Background:**

*Streptococcus agalactiae* (Group B Streptococcus, GBS), a leading cause of sepsis and meningitis in infants, can be transmitted vertically from mother to infant during passage through the birth canal. Detection of GBS colonization in perinatal women is a major strategy for the prevention of postpartum neonatal disease. The U.S. Centers for Disease Control and Prevention recommends that all women undergo vaginal-rectal screening for GBS colonization at 35-37 weeks of gestation. ChromID Strepto B (STRB) is a chromogenic GBS screening media on which GBS colonies appear pink or red, while other bacteria are either inhibited or form colonies in other colors. In this study, we compared STRB with a conventional GBS detection method using 5% sheep blood agar (BA) followed by a selective enrichment broth.

**Methods:**

Anovaginal swabs were collected from 1425 women during weeks 35 to 37 of their pregnancies. The swabs were used to inoculate both STRB and BA plates after enrichment with selective Todd Hewitt Broth (THB). A GBS latex agglutination test was used to confirm the identity of isolates from each plate.

**Results:**

GBS was recovered from 319 (22.4%) samples with one or both media: 318 on STRB compared to 299 using BA. One false negative was observed on STRB, and 20 false negatives were observed on BA. In addition, non-hemolytic GBS was recovered from 19 (6.0%) samples using STRB.

**Conclusions:**

STRB offers effectiveness and convenience over BA for GBS screening in clinical laboratories. STRB produces fewer false negatives, has a higher detection rate and uses a simple color screen that is ideal for technician-level applications. We recommend STRB as the media of choice for GBS screening.

## Background

*Streptococcus agalactiae* (Group B Streptococcus, GBS) is a leading cause of sepsis and meningitis in infants. GBS can be transmitted vertically from mother to infant during passage through the birth canal. Transmission is thought to occur just before or during birth when GBS ascends the genital tract into the amniotic fluid, where it is aspirated or ingested by the infant [1]. Colonization occurs in 3.2-24.3% of pregnant women [2-4]. Prior to 2011, the incidence of early-onset (< 7 days after birth) GBS disease was 0.34-0.37 cases per 1000 births [5], with 22.6% of cases resulting in death or sequelae [6]. In 2011, the U.S. Centers for Disease Control and Prevention (CDC) reported that the incidence of early-onset GBS disease had declined from 1.7 cases per 1000 live births to 0.34-0.37 cases per 1000 live births during the 15 years since the release of prevention guidelines (updated in 2002) [5]. The guidelines recommend perinatal screening of all pregnant women for carriage of GBS at 35-37 weeks of gestation. Vaginal/rectal swabs should be inoculated into a selective broth medium, to aid the recovery of GBS, and incubated 18-24 hours before sub-culturing on sheep blood agar plates or chromogenic agar [5]. The “Guidelines for obstetrical practice in Japan 2011 edition” also recommend that all pregnant women be screened by vaginal/rectal swabs at 33-37 weeks [7], but did not incorporate detailed screening procedures.

ChromID Strepto B (STRB) is a chromogenic agar that was developed to screen for GBS in pregnant women. Using samples from 1425 Japanese women, we evaluated STRB for ease of use and detection rate compared to conventional blood agar. In addition, the occurrence and detection of non-hemolytic GBS was evaluated. To our knowledge, this is the first evaluation of STRB for a large population of Japanese women.

## Methods

### Study design and population

This study was approved by the research ethics committee (Number 264, October 2010) of the Japanese Red Cross Medical Center, Hiroo, Japan. The research ethics committee granted exemption for this study, so that the need for informed consent was waived. Between November 2010 and October 2011, an anovaginal swab was collected from each of 1425 pregnant women at 35-37 weeks of gestation. Sysmex Corporation contributed reagents to the Japanese Red Cross Medical Center.

### Collection and culture of specimens

All specimens were collected using sterile cotton swabs that were submerged in Todd-Hewitt Broth supplemented with 100,000 U/L colistin and 15 mg/L nalidixic acid (THB, Nikken Bio Medical Laboratory). Anovaginal sampling was carried out by rotating a cotton swab against the vaginal wall, then gently rotating the swab to touch the anal crypts. Routine screening of pregnant women for *Streptococcus agalactiae* (GBS) is always performed during the prenatal consultation between 35 and 37 weeks of gestation. All samples were collected by a physician and transported to the Laboratory of Bacteriology Research within two hours. These swabs were taken as part of standard patient care.

**Figure 1:**
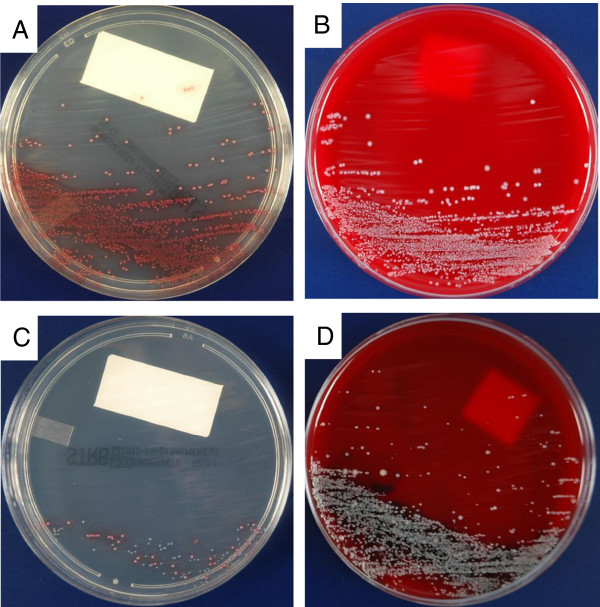
**STRB and BA plates after a 24**-**hours incubation.***Streptococcus agalactiae* (GBS) on STRB **(A)** and blood agar (BA) plates **(B)**; *Enterococcus faecalis* and GBS on STRB **(C)** and BA **(D)**. Pink colonies = GBS; Blue colonies = *E. faecalis*.

The samples were cultured following the CDC recommendations. THB was inoculated with the swab and incubated aerobically at 35°C for 18-24 hours. An aliquot (10 μl) of each enriched THB culture was sub-cultured on chromID Strepto B (STRB, bioMerieux, Marcy l’Etoile, France) and 5% sheep blood agar (BA) plates BBL, BD Diagnostics, Sparks, MD). Both media were incubated at 35°C for 24 hours either aerobically (STRB) or in 5% CO_2_ (BA). The plates were examined for presumptive positives, which were defined as: 1) pale pink to red colonies on STRB (Figure 1A), or 2) white colonies with moist or glistening features and a narrow zone of β-hemolysis (or no hemolysis) on BA (Figure 1B). The BA isolates were used directly in a latex agglutination assay (Prolex Streptococcal Grouping Latex kit, Iwaki, Tokyo, Japan) for GBS identification, whereas the isolates on STRB were first sub-cultured on BA before confirming using latex agglutination (Seroiden Strepto Kit ‘EIKEN’, Eiken Chemical, Tokyo, Japan). The colonies displaying colors other than pink/red were identified using colony characteristics, biochemical reactions, or a combination of these tests. The two different media were assessed by two different institutions in blind tests. GBS on BA was assessed in a hospital, and GBS on STRB was observed by an external clinical laboratory (SRL, Tokyo, Japan). All assessments were performed at each institution by well-trained staff members who were blinded to the results of the other.

## Results

Of the 1425 samples, a total of 319 (22.4%) were positive by at least one of the two media: 318 (22.3%) GBS isolates were recovered from STRB and 299 (21.0%) were recovered from BA. Although STRB recovered more GBS-positive isolates, it produced fewer false negatives, with only one compared to 20 for BA. Non-GBS colonies on STRB, displaying colors other than pink/red, were identified as *Enterococcus* spp., *Streptococcus* spp., coagulase negative *Staphylococcus* spp., *Corynebacterium* spp., and *Lactobacillus* spp. Of the 19 (6.0%) non-hemolytic GBS isolates, 13 were recovered from GBS-positive samples on both STRB and BA and six from GBS-positive samples only on STRB. None of the non-hemolytic GBS isolates grew on BA only.

## Discussion

One of the most important requirements for a screening protocol is the minimization of false negatives which, in this case, can produce a high risk of neonatal GBS infection. Blood agar is widely used around the world for the culture of hemolytic Group B Streptococci (GBS). Two or more species of bacteria in recto-vaginal samples can make detection of GBS difficult. In our study, only one positive result that was missed following the 24-hour incubation on STRB eventually displayed pink colonies upon a 48-hour incubation. This result shows a trend that is similar to other studies. Tazi et al. [8] and Robin et al. [9] found that both media have a sensitivity of 100% following a 48- to 72-hour incubation after enrichment. Although Aila et al. [10] also reported 100% sensitivity for STRB, blood agar was much lower at 78% after 24-hour post-enrichment incubations. A comparison of detection rates indicates that STRB is a good medium with better sensitivity than that of BA.

In addition to the detection rate, the STRB detection mechanism confers three additional benefits. The first advantage is the easy visual identification of GBS compared with blood agar, even when the specimen contains a low level of GBS or when GBS in mixed with other microorganisms. Bacteria other than GBS display different colors or are inhibited by antibiotics. Therefore, GBS stands out against background flora (Figure 1C, D).

The second advantage is the detection of non-hemolytic GBS. Non-hemolytic and non-pigmented GBS strains have been isolated from pregnant women at a level ranging from 1 to 4% [11-17]. Tazi et al. [8] found that non-hemolytic and non-pigmented strains are responsible for meningitis and sepsis in newborns. Miranda et al. [18] also reported that non-hemolytic GBS can cause endocarditis. Because these reports suggest that infection with non-hemolytic GBS can cause neonatal GBS disease, non-hemolytic colonies must also be tested for the presence of GBS. STRB can identify non-hemolytic GBS with the same simple color scheme that is used for hemolytic strains. In this study, of the 319 GBS isolates, 19 were non-hemolytic. Among the 20 false negatives on BA plates were 6 non-hemolytic GBS samples. These data indicate that STRB is more suited than BA for the detection of non-hemolytic GBS strains.

The third advantage is that STRB plates are incubated in an aerobic environment, whereas BA is generally incubated in 5% CO_2_.

Although the positive rate of the STRB screen is excellent, there are some disadvantages. *Streptococcus porcinus* and *Streptococcus anginosus* give false positive results on STRB [8,10]. We also found few *Enterococcus* spp. and *Streptococcus* spp. strains on STRB in this study (no statistical data). The target organisms are characterized by enzyme systems that metabolize the substrate to release the chromogen, which enables the organisms to display a typical color. STRB includes three chromogenic substrates, and three enzymes (phosphatase, esterase and β-cellobiosidase) react with these substrates such that the color of the organisms results from the combination of these three substrates. Each substrate gives a pink, blue, and purple color for phosphatase activity, esterase activity, and β-cellobiosidase activity, respectively. GBS are colored by only one of these three substrates, and thus show only one color. On the other hand, other species are colored by one to three enzymatic reactions so that various color combinations and variable colors can be seen. A given occurrence or enzymatic activity for each substrate can differ between organisms. In our study, some *Streptococcus* spp. other than GBS and *Enterococcus* spp. strains showed pink colonies, while others showed blue, white and purple colonies. *Enterococcus* spp. and *Streptococcus* spp. on STRB was sub-cultured on blood agar. *Enterococcus* spp. was identified by Gram stain, 6.5% NaCl, bile esculin and EF agar. *Streptococcus* spp. was identified by Gram stain, 6.5% NaCl, bile esculin, EF agar, hemolysis and Optochin test. Because false positives are a possibility, an additional confirmatory test using a biochemical or immunological assay, as recommended by the manufacturer, should be performed on each isolate. STRB contains light-sensitive components and should not be exposed to light except when necessary during the inoculation and reading steps. These precautions may make the test more complicated for laboratory technicians.

BA is a non-selective conventional agar [19]. The absence of antibiotics in the media allows most organisms to grow easily, especially gram positive organisms, since the growth of gram negative organisms is inhibited during enrichment. Therefore, large numbers of non-GBS organisms may make it difficult to isolate GBS, especially if those organisms produce large β-hemolytic zones. In this study, the 20 false negatives obtained on BA grew as small colonies on STRB or were mixed with organisms that had large β-hemolytic zones. A greater level of expertise is needed to identify GBS on BA from characteristics alone, increasing the possibility of variability in interpretation between technicians.

## Conclusions

The detection rate of STRB with enrichment was higher than BA with enrichment. Moreover, STRB has the advantages of easy detection and decreased risk of variability in interpretation. STRB is also particularly useful for the detection of non-hemolytic GBS colonies. Extending incubation time past 24 hours decreases the likelihood of identifying false negatives.

## Competing interests

DF works for Sysmex Corporation, which provided reagents for the study. The other authors declare that they have no competing interests.

## Authors’ contributions

TM, YK, TS, KI and MI performed the laboratory work. AM was responsible for specimen collection. TM, MF, DF participated in study design, data analysis and interpretation, and manuscript preparation. All authors read and approved the final manuscript.

## Pre-publication history

The pre-publication history for this paper can be accessed here:

http://www.biomedcentral.com/1471-2334/14/46/prepub
